# Forme pseudotumorale de la tuberculose pulmonaire et les difficultés diagnostic: à propos d’un cas

**DOI:** 10.11604/pamj.2013.14.81.1629

**Published:** 2013-03-03

**Authors:** Aziz Ouarssani, Fouad Atoini, Rafik Reda, Fatima Ait Lhou, Mustapha Idrissi Rguibi

**Affiliations:** 1 Service de pneumologie, hôpital militaire Moulay Ismail, Meknès, Maroc; 2 Service de chirurgie thoracique, hôpital militaire Moulay Ismail, Meknès, Maroc; 3 Service de neurologie, hôpital militaire Moulay Ismail, Meknès, Maroc

**Keywords:** Tuberculose, Poumon, Pseudotumorale, Antibacillaire, Tuberculosis, lung, pseudotumoral, antibacillary

## Abstract

La tuberculose pulmonaire est un problème majeur de santé publique. Dans sa forme commune, le diagnostic est habituellement aisé, mais elle peut se présenter sous une forme trompeuse et entrainer un retard diagnostic et thérapeutique. Nous rapportons le cas d’un patient âgé de 25ans, étudiant, sans antécédents pathologiques particuliers, hospitalisé dans notre formation pour un syndrome bronchique trainant avec altération de l’état général. L’examen clinique pleuropulmonaire est normal, l’examen des aires ganglionnaires trouve une adénopathie sus claviculaire droite, la radiographie thoracique de face objective une opacité hilaire droite hétérogène à contours irréguliers, la TDM thoracique retrouve un processus lésionnel tissulaire du lobe supérieur droit qui s’étend vers le médiastin et englobe partiellement la veine cave supérieure avec une adénopathie latérotracheale droite nécrosée. La fibroscopie bronchique objective un élargissement des éperons intersegmentaires du lobe supérieur droit, les biopsies réalisées avec étude histologique sont non concluantes. Les recherches de BK dans les expectorations et dans le liquide d’aspiration bronchique sont negatives. L’IDR à la tuberculine est à 15mm. C’est la ponction transparietale scannoguidée avec étude anatomopathologique qui confirme le diagnostic de tuberculose caseofolliculaire. La sérologie VIH est négative. Le diagnostic de tuberculose pulmonaire pseudotumorale chez un immunocompétent a été retenu et le patient a été mis sous traitement antibacillaire (régime standard national Marocain) par rifampicine, isoniazide, pyrazinamide et éthambutol pendant 6 mois avec évolution clinique et radiologique favorable. La tuberculose pulmonaire ne cesse de tromper le clinicien par son polymorphisme clinique et radiologique, elle doit être évoquée devant toute atteinte pulmonaire d’allure tumorale pour permettre une prise en charge précoce de la maladie.

## Introduction

La tuberculose pulmonaire est caractérisée par une grande diversité de son expression clinique et radiologique. La forme pseudotumorale est rare, peut simuler un cancer broncho-pulmonaire par la présentation clinique, radiologique et/ ou endoscopique.

## Patient et observation

**Figure 1:**
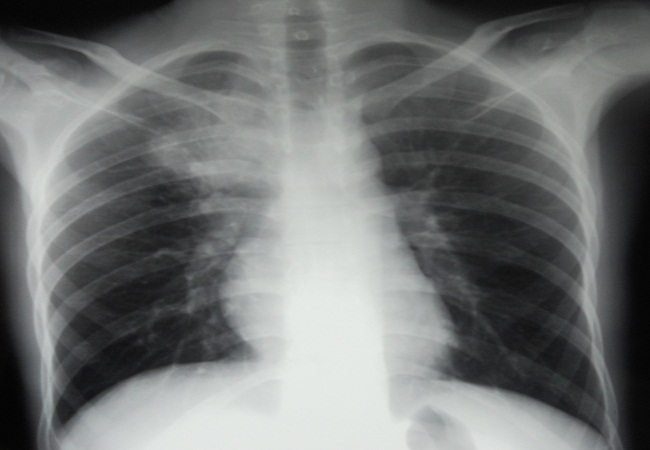
La radiographie thoracique de face objective une opacité hilaire droite hétérogène à contours irréguliers

**Figure 2:**
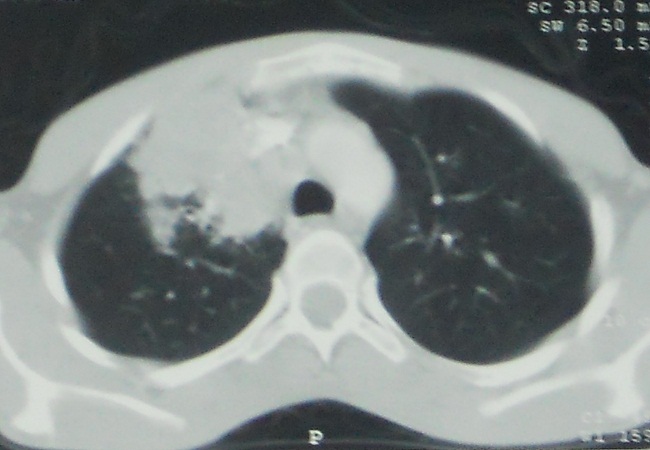
La TDM thoracique retrouve un processus lésionnel tissulaire du lobe supérieur droit qui s’étend vers le médiastin et englobe partiellement la veine cave supérieure avec une adénopathie latérotracheale droite nécrosée faisant évoquer un processus d’allure tumoral

Mr A.B, âgé de 25 ans, étudiant, sans antécédents pathologiques notables et sans habitudes toxiques, hospitalisé pour un syndrome bronchique trainant fait de toux ramenant des expectorations purulentes évoluant depuis 5 mois, dans un contexte de fièvre et d’amaigrissement chiffré à 10 kilogramme. L’examen à l’admission trouve un patient en assez bon état général, pesant 46 kg pour une taille de 1,75m, febrile à38, 5° l’examen pleuropulmonaire est sans particularité, l’examen des aires ganglionnaires trouve une adénopathie sus claviculaire droite, fixe par rapport au plan profond. La radiographie thoracique de face objective une opacité hilaire droite hétérogène à contours irréguliers (Figure 1), la TDM thoracique retrouve un processus lésionnel tissulaire du lobe supérieur droit qui s’étend vers le médiastin et englobe partiellement la veine cave supérieure avec une adénopathie latérotracheale droite nécrosée faisant évoquer un processus d’allure tumoral (Figure 2). La fibroscopie bronchique objective un élargissement des éperons intersegmentaires du lobe supérieur droit, les biopsies réalisées avec étude histologique sont non concluantes. Les recherches de BK dans les expectorations et dans le liquide d’aspiration bronchique sont negatives.L’IDR à la tuberculine est à 15mm. Le bilan biologique trouve une CRP à 93,61 mg/l, une hyperleucocytose à 12450/Ul à polynucléaire neutrophile (69,5%).La sérologie VIH est négative. Une biopsie de l’adénopathie sus claviculaire droite a été faite, et l’étude histologique objective une lymphadénite chronique réactionnelle sans signe d’inflammation spécifique.

**Figure 3:**
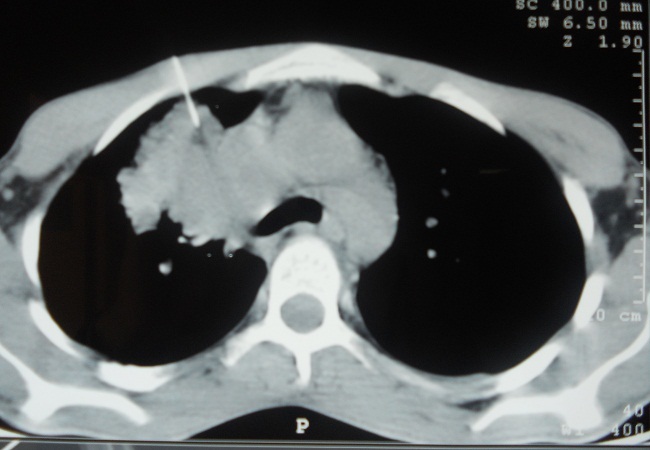
Coupe scannographique avec ponction transparietale scannoguidée

**Figure 4:**
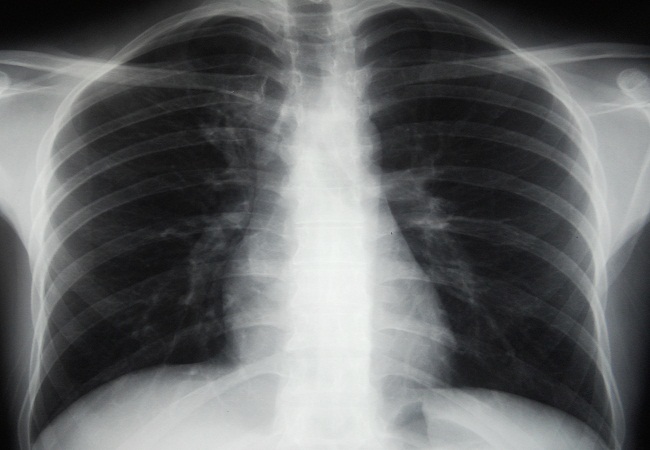
La radiographie thoracique de contrôle à la fin du traitement (6éme mois) objective une régression complète des lésions radiologiques

Une ponction transparietale scannoguidée a été faite (Figure 3) et l’étude anatomopathologique confirme le diagnostic de tuberculose caseofolliculaire. Le diagnostic de tuberculose pulmonaire pseudotumorale chez un patient immunocompétent a été retenu et le patient a été mis sous traitement antibacillaire (régime standard national Marocain) par rifampicine, isoniazide, pyrazinamide et éthambutol pendant 6 mois. L’évolution est marquée par une amélioration clinique, une prise de poids, et la radiographie thoracique de contrôle à la fin du traitement objective une régression compléte des lésions radiologiques (Figure 4).

## Discussion

Le diagnostic de la tuberculose pulmonaire commune est habituellement facile, il est orienté par les données cliniques et radiologiques et confirmé par la positivité des prélèvements bactériologiques. Dans sa forme pseudotumorale, le tableau radioclinique est atypique et trompeur [1]. En effet, la moyenne d’âges est plus élevée, elle varie entre 39 et 56,8 ans selon les séries, la prédominance masculine est nette ce qui fait évoquer plutôt un cancer broncho-pulmonaire.

La tuberculose pseudotumorale survient souvent chez les patients infectés par le VIH, elle est rare chez les immunocompétents et ne représente que 4,3% dans l’étude de Cherlan et al [2]. Le diagnostic est souvent tardif témoignant des difficultés diagnostiques, il varie de 4 à 10 semaines selon les auteurs, la symptomatologie fonctionnelle est non spécifique, la toux, la douleur thoracique, l’hémoptysie et l’altération de l’état général, signes les plus fréquents, orientent plutôt chez le sujet âgé vers une pathologie néoplasique. Les prélèvements bactériologiques, négatifs à l’examen direct, sont rarement positifs aux cultures. Cela est lié au caractère solide et mal oxygéné des lésions caséeuses dans la tuberculose pseudotumorale [3, 4].

L’aspect tomodensitométriques est souvent évocateur de malignité en montrant des lésions de densité tissulaire ou des condensations parenchymateuses systématisée avec des limites spéculées, la prise de contraste annulaire des lésions parenchymateuse et des ganglions, les calcifications et l’aspect d’arbre en bourgeon fortement évocateur de la tuberculose ne sont pas spécifiques et sont rarement retrouvés. Comme dans la tuberculose pulmonaire maladie, les lésions prédominent au niveau des segments apicaux et dorsaux des lobes supérieurs et les segments apicaux des lobes inferieurs avec une prédominance du coté droit[5].

La fibroscopie bronchique montre un aspect ulcéroinfiltrés et surtout bourgeonnant renforçant la suspicion de carcinome bronchique, les biopsies bronchiques confirment le diagnostic en cas de tuberculose bronchique mais dans les formes sans lésion endobronchique, la présence de secrétions blanchâtre sous muqueuses adhérentes correspondant à du caséum et / ou une muqueuse grisâtre ou anthracosique chez un jeune patient non fumeur doit inciter à rechercher une tuberculose. D’autres moyens diagnostiques plus invasifs s’avèrent indispensables, telles que la biopsie transparietale et la biopsie chirurgicale avec étude de la pièce opératoire [6].

Cette difficulté diagnostique de la tuberculose pseudotumorale est à l’origine d’un retard diagnostique considérable, et varie de 30 à 70 jours selon les auteurs, dans notre cas il est estimé à 28 jours. Ce retard est expliqué par la négativité habituelle des prélèvements bactériologique, la culture très lente du BK et la difficulté des prélèvements biopsiques dans les formes sans lésions endobronchique [7]. Contrairement aux difficultés diagnostiques, le traitement de la tuberculose pseudotumorale est souvent facile, et est basé sur les antituberculeux aux posologies et durées usuelles. L’évolution est favorable, cependant certaines complications peuvent survenir: à type de sténose, de bronchectasie et de broncholithiase et nécessiter des traitements instrumentaux par le laser, la cryothérapie, et la dilatation par ballonnet ou un traitement chirurgical [8].Chez notre patient, l’évolution est favorable sous traitement antibacillaire avec nettoyage radiologique quasi complet.

## Conclusion

La tuberculose pseudotumorale est une forme rare de tuberculose dont le diagnostic positif est difficile du fait d’un tableau radioclinique atypique faisant évoquer un cancer bronchique, et la négativité habituelle des prélèvements bactériologiques à l’origine d’un retard diagnostic.
